# Mechanistic insights into resistance mechanisms to T cell engagers

**DOI:** 10.3389/fimmu.2025.1583044

**Published:** 2025-04-22

**Authors:** Linlin Cao, Gabrielle Leclercq-Cohen, Christian Klein, Antonio Sorrentino, Marina Bacac

**Affiliations:** ^1^ Roche Innovation Center, Zürich, Switzerland; ^2^ Curie.Bio, Boston, MA, United States; ^3^ Roche Innovation Center, Basel, Switzerland

**Keywords:** resistance mechanism, T cell engager, intrinsic mechanism, tumor microenvironment, T cell function

## Abstract

T cell engagers (TCEs) represent a groundbreaking advancement in the treatment of B and plasma cell malignancies and are emerging as a promising therapeutic approach for the treatment of solid tumors. These molecules harness T cells to bind to and eliminate cancer cells, effectively bypassing the need for antigen-specific T cell recognition. Despite their established clinical efficacy, a subset of patients is either refractory to TCE treatment (e.g. primary resistance) or develops resistance during the course of TCE therapy (e.g. acquired or treatment-induced resistance). In this review we comprehensively describe the resistance mechanisms to TCEs, occurring in both preclinical models and clinical trials with a particular emphasis on cellular and molecular pathways underlying the resistance process. We classify these mechanisms into tumor intrinsic and tumor extrinsic ones. Tumor intrinsic mechanisms encompass changes within tumor cells that impact the T cell-mediated cytotoxicity, including tumor antigen loss, the expression of immune checkpoint inhibitory ligands and intracellular pathways that render tumor cells resistant to killing. Tumor extrinsic mechanisms involve factors external to tumor cells, including the presence of an immunosuppressive tumor microenvironment (TME) and reduced T cell functionality. We further propose actionable strategies to overcome resistance offering potential avenues for enhancing TCE efficacy in the clinic.

## 1 Introduction

**Figure 1:**
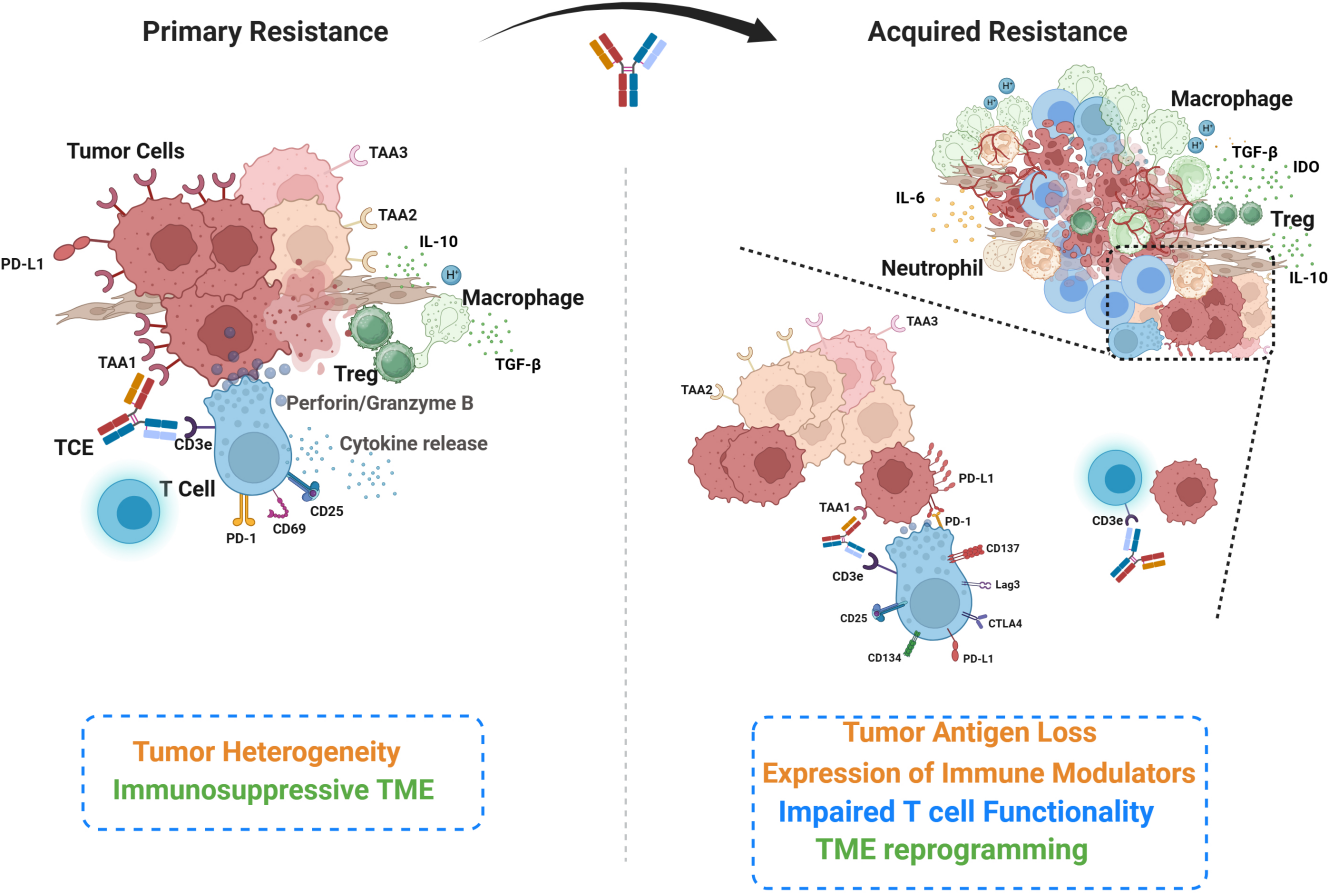
Overview of the key cellular and molecular players underlying primary and acquired resistance to TCEs. Primary resistance (left) is characterized by inherent tumor heterogeneity, encompassing tumor cell clones expressing varied tumor-associated antigens, and components of an immunosuppressive tumor microenvironment (TME). This panel also illustrates the initial phases of T cell engagement and activity, including synapse formation and cytokine release. The acquired resistance (right) develops during the course of T cell engager (TCE) treatment. While TCE may eliminate some antigen-expressing cancer cells, other cancer cells may undergo antigen loss for escape. Expression of immune modulators (eg. PD-L1) on cancer cells can suppress intra-tumor T cells and affect their functionality. Immunosuppressive cells infiltrate and reprogram the TME following TCE treatment. The zoomed inset on the left illustrates T cell activation following TCE treatment, characterized by the upregulation of activation and exhaustion markers that modulate T cell function and suggest opportunities for combination therapies. The inset on the right depicts tumor antigen loss, which disrupts TCE-mediated T cell–tumor cell engagement, resulting in impaired cytotoxicity and tumor immune escape.

**Figure 2:**
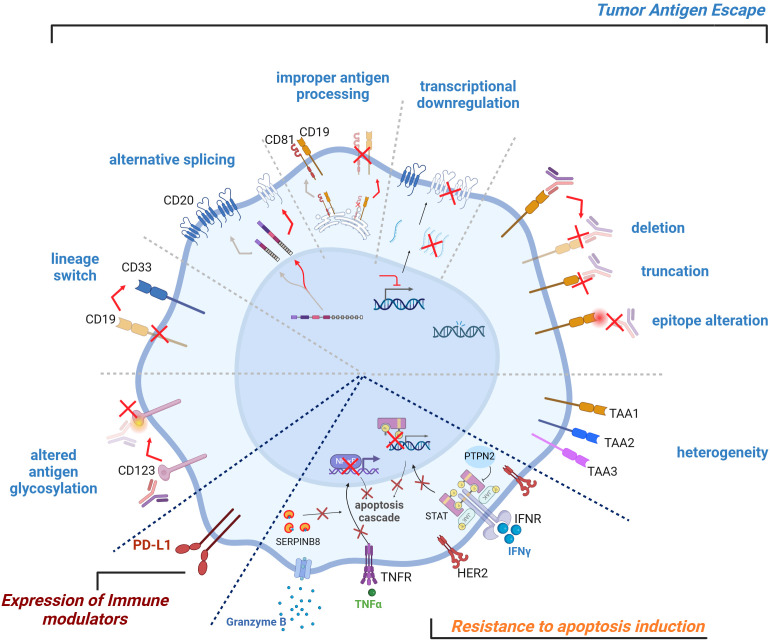
Tumor-intrinsic TCE resistance mechanisms. Tumor-intrinsic TCE resistance mechanisms can affect the efficacy of T cell engagers. Key factors include tumor antigen escape, expression of immune modulators, resistance to apoptosis induction. Heterogeneous tumor antigen expression, genetic aberrations, transcriptional downregulation, improper antigen processing and/or presentation, alternative splicing, lineage switch, altered antigen glycosylation can all lead to tumor cell antigen escape. Resistance can also arise from the expression of checkpoint inhibitory ligands on the tumor cell surface, which can dampen T cell activity. Conversely, the lack of co-stimulatory signals on tumor cells can hinder effective T cell activation. Finally, tumor cells can develop insensitivity towards T cell-mediated cytotoxicity and subsequent apoptosis induction, further contributing to resistance. TCE, T Cell Engager.

**Figure 3:**
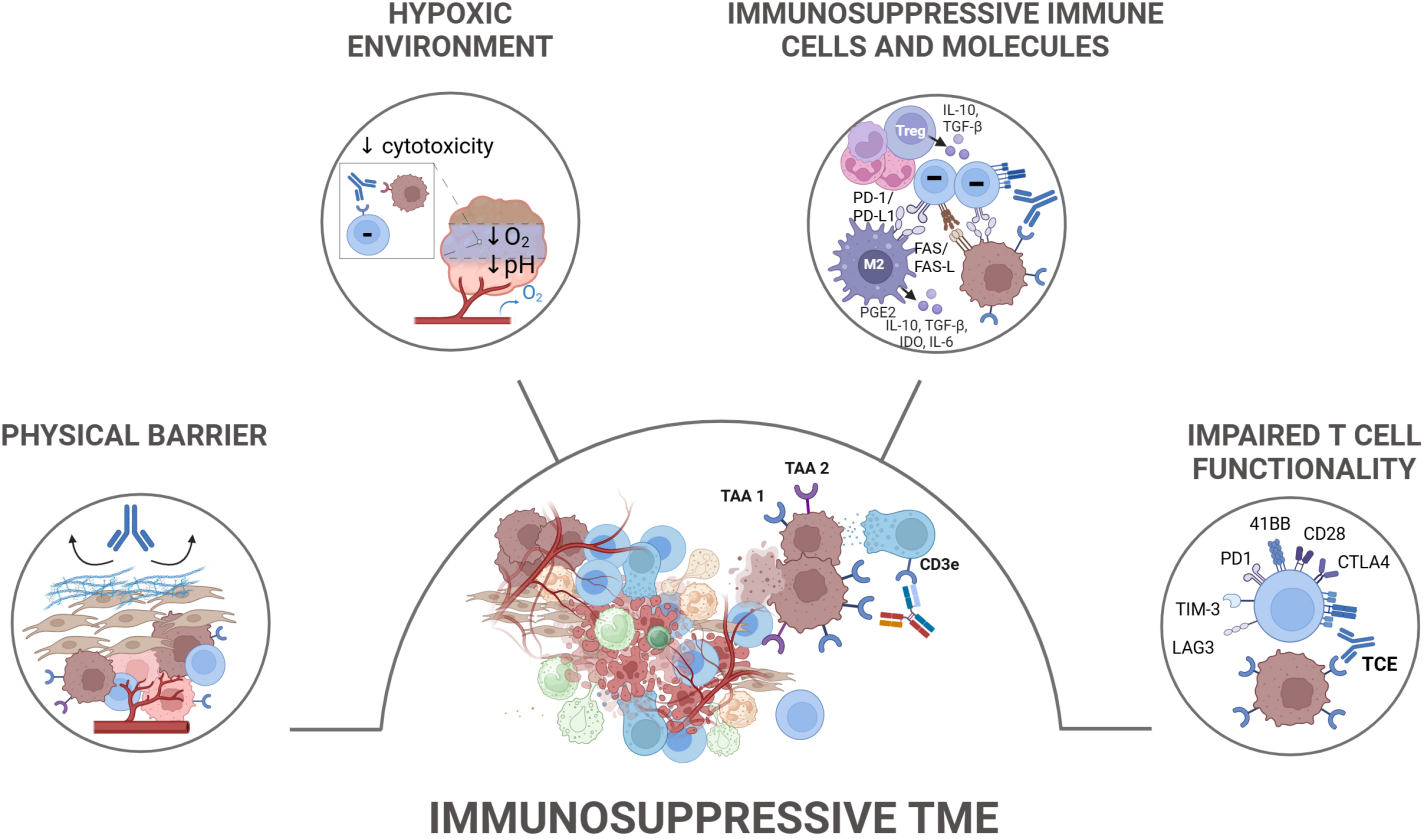
Tumor-extrinsic TCE resistance mechanisms. Tumor-extrinsic mechanisms contributing to resistance to TCEs primarily involve the immunosuppressive tumor microenvironment (TME) and T cell-intrinsic dysfunction. The immunosuppressive TME is characterized by the presence of various cellular components such as regulatory T cells (Tregs), tumor-associated macrophages (TAMs), myeloid-derived suppressor cells (MDSCs), and cancer-associated fibroblasts (CAFs), which collectively suppress T cell functionality. These cells secrete inhibitory cytokines, such as IL-10 and TGF-β, metabolically starve effector T cells, and express inhibitory ligands. In solid tumors, the TME also presents physical barriers like dense stroma and vasculature that limit TCE penetration and T cell infiltration, in addition to metabolic features including nutrient starvation, hypoxia, and acidity. T cell-intrinsic dysfunction, indicated by the upregulation of inhibitory receptors such as PD-1, TIM-3, and TIGIT, further contributes to resistance. TAA, Tumor Associated Antigen; TCE, T Cell Engager.

T cell engagers (TCEs) represent a transformative advancement in the treatment landscape of cancer patients with B and plasma cell malignancies. TCEs are designed to engage any type of T cells via the CD3ϵ subunit of the T cell receptor (TCR) complex and bring them in close proximity of tumor cells, enabling the formation of immunological synapses and subsequent tumor cell killing, effectively bypassing the necessity for antigen-specific T cell recognition (1–3). Over more than three decades of research, numerous TCE formats have been developed and are currently undergoing preclinical and clinical evaluation. While the primary focus of this review is on resistance mechanisms to TCEs, we acknowledge the importance of TCE format and antibody design. Therefore, we refer the reader to several excellent reviews that comprehensively cover the evolution of TCE constructs and platforms (2, 4–9). Several TCEs have been approved for the treatment of hematological malignancies, including blinatumomab (CD19 x CD3 TCE) (10), teclistamab (BCMA x CD3 TCE) (11), mosunetuzumab (CD20 x CD3 TCE) (12), epcoritamab (CD20 x CD3 TCE) (13), glofitamab (CD20 x CD3 TCE) (14), talquetamab (GPRC5D x CD3 TCE) (15), elranatamab (BCMA x CD3 TCE) (16), and odronextamab (CD20 x CD3 TCE) (17). In solid tumors, the TCR-based gp100-peptide MHC specific T cell engaging ImmTAC tebentafusp was approved for the treatment of metastatic uveal melanoma (18), and tarlatamab (DLL3 x CD3 TCE) for the treatment of small cell lung cancer (SLCL) (19, 20). In general, the development of TCEs for solid tumor indications has proven to be more complex and challenging, attributed to the lack of highly tumor specific antigens absent in normal tissues, higher tumor heterogeneity (21), and a more immunosuppressive TME (22). Furthermore, high mutational burden, clonal evolution, epigenetic modifications collectively contribute to the establishment of resistance mechanisms that hamper TCE efficacy (23, 24). Resistance to TCEs can be either primary (e.g. existing prior to treatment), leading to tumor refractoriness and lack of response to TCE treatment, or acquired (treatment-induced), which occurs upon consecutive treatments with TCEs (**Figure 1**). Both types of resistance restrict the benefit to a subset of patients, affect response durability and pose challenges to clinical development. At the cellular and molecular level, TCE-related resistance mechanisms differ from resistance to targeted therapies directed to tumor drivers, which often arise through mutational escape of the specific molecular target. Instead, TCE resistance is complex, multifaceted, and not yet fully understood, involving both factors intrinsic within tumor cells and extrinsic influences from the tumor microenvironment. Tumor cell intrinsic factors include heterogenous tumor antigen expression, expression of checkpoint inhibitory ligands, lack of co-stimulatory signals, and activation of signaling pathways that render cancer cells resistant to apoptosis (**Figure 2**). Conversely tumor cell extrinsic resistance mechanisms are primarily driven by an immunosuppressive TME, which includes cellular and secreted factors that cumulatively suppress T cell functionality and their ability to perform TCE-mediated cytotoxic activity (**Figure 3**).

As resistance mechanisms present a significant barrier to the clinical efficacy and advancement of TCEs, a comprehensive understanding of these processes is essential for the rational design of next-generation therapeutics and the development of novel combinatorial strategies to overcome resistance. In the current review, we systematically examine both established and emerging resistance mechanisms to TCE therapies, integrating current evidence with proposed hypotheses. Furthermore, we outline actionable strategies to overcome these barriers, aiming to optimize TCE efficacy, enhance durability of response, and ultimately improve clinical outcomes in patients receiving TCE-based therapies (**Table 1**).

**Table 1:** Overview of resistance mechanisms to TCEs and mitigation strategies.

	Resistance mechanisms	Detailed category	Mitigation
Tumor-intrinsic resistance mechanisms	Tumor Antigen-related mechanisms	Tumor antigen heterogeneity	• Tumor debulking with chemotherapy, ADCs, TKIs • Targeting of multiple tumor antigens or alternating tumor antigen targeting • Restoration of antigen expression or prevention of antigen loss
		Tumor antigen loss	
		Lineage switch	
		Gene expression shift	
		Post-translational modification	
	Tumor cell expression of immunomodulatory molecules	Expression of checkpoint inhibitors, loss of costimulation (CD58), post-translational modification	Combination with CPIs (eg. αPD-1, αPD-L1)
	Tumor cell resistance to apoptosis induction	Interference with granzyme/perforin-mediated apoptosis Altered response to TNF-α and IFN-γ	Tumor debulking with chemotherapy, ADCs, TKIs
Tumor-extrinsic resistance mechanisms	Immunosuppressive tumor microenvironment	Physical barriers	• Myeloid cell depletion or engagement • Treg depletion • NK cell engagement
		Hypoxic environment	
		Immunosuppressive immune cells and molecules	
		Impaired T cell functionality	• Costimulator combination (4-1BB/CD137, CD28 agonists) • Treatment free intervals • CPI combination

## 2 Tumor intrinsic resistance mechanisms

### 2.1 Tumor antigen-related resistance mechanisms

#### 2.1.1 Tumor antigen heterogeneity

Tumor heterogeneity is a fundamental challenge in oncology, encompassing genetic, phenotypic, and microenvironmental variability within and across tumors (25–27). This complexity influences disease progression, therapeutic response, and resistance mechanisms (21, 24). One key aspect is tumor antigen heterogeneity, which impacts the efficacy of targeted therapies and immunotherapies, including TCEs, monoclonal antibodies, and chimeric antigen receptor (CAR) T cell therapy (24, 28–30).

In solid tumors, antigen heterogeneity arises due to clonal evolution, epigenetic modifications, and selective pressures from the TME (31). This results in differential antigen expression across tumor subpopulations, with some cells expressing high levels of target antigens while others exhibiting low or undetectable levels, leading to immune escape and therapy resistance. Furthermore, spatial heterogeneity contributes to differential antigen accessibility, particularly in tumors with extensive stromal components or poor vascularization.

In contrast, hematologic malignancies often exhibit more uniform antigen expression across malignant cells due to their clonal origin. However, dynamic antigen modulation, shedding, and immune pressure-driven loss of antigen expression can still occur, affecting the efficacy of TCEs and other targeted immunotherapies. Additionally, antigen density and lineage plasticity in hematologic cancers may influence treatment outcomes, particularly in relapsed/refractory (R/R) settings, where tumor cells downregulate or alter antigen presentation to evade immune cell attack (32, 33).

Preclinical experiments showed that TCE-mediated killing may depend on the level of antigen expression. However, due to the high potency of T cell killing, TCEs can generally kill target cells that express low antigen levels e.g. tens to hundreds or thousands copies/cell, as shown for peptide-MHC targeting TCEs or other potent TCEs targeting classical tumor antigens (34–37). Therefore, in the context of TCEs, a strict threshold for the target antigen expression level required for activity has not been consistently identified. In line with this, preclinical studies found no correlation between CD20 expression and glofitamab activity. *In vitro* cytotoxicity was observed across various tumor cell lines, regardless of CD20 expression levels, with strong potency even in cell lines expressing low CD20 receptor levels (35, 36). In line with preclinical findings, the baseline level of CD20 expression did not correlate with clinical response to CD20-targeted TCEs in Non-Hodgkin’s Lymphoma (NHL), including glofitamab (38), mosunetuzumab (39), and odronextamab (40), even though a most recent report showed that reduced CD20 expression in patients with B-cell neoplasms was associated with inferior survival when treated with CD20 x CD3 TCEs (Emil Ramsø Kyvsgaard , Reduced CD20 Expression yields Inferior Survival in Patients with B Cell Lymphoma Treated with CD20xCD3 Antibodies, Blood Neoplasia 2025). Similarly, multiple myeloma baseline tumor BCMA and GPRC5D expression did not significantly differ between responders and non-responders to teclistamab and talquetamab in multiple myeloma (41). Likely in these cases even low target expression was above the minimal expression levels required for killing. In contrast, *ex vivo* cytotoxicity using primary tumor biopsies showed a correlation between higher BCMA expression and greater sensitivity to teclistamab compared to talquetamab. Similarly, a higher GPRC5D expression correlated with higher sensitivity to talquetamab compared to teclistamab (41), pointing to a discrepancy between the *ex vivo*/*in vitro* studies and clinical findings.

In the case of solid tumors, *in vitro* preclinical studies showed that cibisatamab’s (CEA x CD3 TCE) activity (a low potency TCE) strongly correlated with CEA expression, with higher potency observed in highly CEA-expressing tumor cells and a threshold of approximately 10,000 CEA-binding sites per cell, which allowed distinguishing between high- and low-CEA-expressing tumor and primary epithelial cells, respectively (42). Furthermore, genetic factors did not affect *in vitro* cytotoxic activity, confirming that CEA expression level was the strongest predictor of cibisatamab’s *in vitro* activity. Further experiments conducted using patient derived colorectal cancer organoids also highlighted that heterogeneity and plasticity of CEA expression conferred low cibisatamab sensitivity (31). In the recent Phase 1 trial with cibisatamab with or without the anti-PD-L1 antibody atezolizumab, no clear correlation between CEA expression and clinical response to cibisatamab was observed in the overall population (43). However, a more granular exploratory analysis of the cibisatamab plus atezolizumab cohort found that all four patients with a confirmed partial response (PR) had high expression of CEACAM5 mRNA (43). Unfortunately, given the small cohort size, it is difficult to conclude at the moment about the correlation between clinical activity of cibisatamab and patient tumor CEA expression level.

In conclusion, given that TCEs depend on tumor antigen binding for activity, it is reasonable to believe that the level and heterogeneity of tumor antigen expression influences treatment outcomes. Preclinical models, including *in vitro* studies with tumor cell lines and organoids, as well as *ex vivo* analyses of patient tumor samples, yielded mixed results - showing a clear correlation for some TCEs, while no correlation was observed for others. At the same time, published clinical findings in heme and solid tumors outlined a lack of correlation between the baseline (pre-treatment) tumor antigen expression and clinical response to TCEs, with responses occurring even in patients whose tumors exhibited low or undetectable antigen expression by immunohistochemistry (IHC). One key consideration is that IHC-negative tumor biopsies do not necessarily indicate a complete absence of target antigen expression. Even tumors classified as IHC-negative can still express up to 2000 target molecules per cell. Given the high potency of TCEs, even such low antigen densities may allow for effective T cell engagement and tumor cell killing. Furthermore, tumor heterogeneity, antigen shedding, internalization, differential antigen expression between primary and metastatic lesions, and variability in antigen accessibility further complicate the direct translation of preclinical findings to clinical responses. Similarly, antigen accessibility varies across tumor types due to factors such as stromal architecture and vascularization, affecting TCE penetration and efficacy.

These findings highlight the inherent limitations of preclinical models in predicting clinical efficacy and point to reconsidering rigid antigen expression thresholds as definitive predictive biomarkers for TCE efficacy in clinical settings. More quantitative and dynamic antigen assessment methods, such as mass spectrometry-based proteomics, digital spatial profiling, or single-cell antigen quantification, may provide a more accurate representation of antigen availability for therapeutic engagement.

#### 2.1.2 Tumor antigen loss

While data for antigen expression and response are variable, true loss of surface tumor antigen expression (in case of TCEs targeting surface tumor antigens), or loss of tumor antigen presentation by class I Human Leukocyte Antigens (HLA-I) (in case of TCE targeting intracellular antigens), are critical (treatment-induced) resistance mechanisms that cancer cells acquire to evade immune detection and elimination. Different mechanisms have been reported to be responsible for the loss of different tumor antigens in preclinical models and clinics, including genomic aberrations, transcriptional downregulation and epigenetic silencing, improper surface antigen processing and/or presentation, alternative splicing, and are briefly summarized below (**Figure 2**).

##### Genomic aberrations

Genetic alterations, including gene mutations, are well-documented mechanisms leading to tumor antigen loss. Resistance to blinatumomab represents a clinical challenge for the treatment of relapsed or refractory B-cell acute lymphoblastic leukemia (B-ALL) in adults and children and is often associated with the loss of CD19 antigen expression or mutations affecting the binding epitope. Whole exome sequencing of genomic DNA revealed that CD19 mutations resulted in a truncated form of the antigen, which is no longer recognized by blinatumomab (44). In addition, CD19-mutant allele-specific expression could lead to loss of CD19 expression (44).

Similarly, loss of CD20 expression is a mechanism of resistance following treatment of patients with B-cell malignancies with CD20-targeting TCEs. Whole exome sequencing (WES) revealed a variety of genomic aberrations encompassing the *MS4A1* gene (encoding the CD20 protein) in patients who relapsed to mosunetuzumab (39, 45), ordronextamab (40), and glofitamab treatment (46). These variants led to either a loss of CD20 expression due to truncating or frameshift mutations, or to changes in the targeting epitope, preventing TCE binding and subsequent activity.

Furthermore, Whole Genome Sequencing (WGS) and Copy Number Variation (CNV) analysis revealed that in a cohort of relapsed refractory multiple myeloma patients treated with BCMA-targeted TCEs (teclistamab, elranatamab), one relapsed patient harbored biallelic deletion of *TNFRSF17* gene (encoding BCMA protein), as well as five patients harboring non-truncating, missense mutations or in-frame deletions in the extracellular domain of BCMA, which negatively affected the binding and subsequently efficacy of BCMA-targeted TCEs (47, 48). WGS analysis of tumor biopsies of patients treated with talquetamab revealed GPR5CD loss due to genetic mutations in relapsed patients (47, 49).

##### Antigen downregulation

Downregulation of target antigen at the transcription level has been observed in patients with CD20 loss when treated with mosunetuzumab (39), and in patients with CD19 loss when treated with blinatumomab (44). Additionally, single-nucleus multi-omic analysis of the MYRACLE (Myeloma Resistance And Clonal Evolution) cohort revealed a lack of *GPRC5D* transcript in two relapsed patients due to long-range epigenetic silencing of *GPRC5D* locus (49). In a preclinical study of acquired resistance a reduction of CEA antigen expression was observed following treatment with CEA-targeting TCEs, but not in tumors treated with a HER2-targeting TCE (50). The proposed mechanism for this reduction was an indirect methylation effect on the *CEACAM5* locus (50), whereas HER2 expression was kept constant as it is essential for tumor cell proliferation and survival.

##### Improper surface antigen processing and/or presentation

Surface antigen expression requires proper intracellular protein maturation and trafficking. Interestingly, CD81, a chaperone protein regulating CD19 processing from the endoplasmic reticulum (ER) and Golgi Apparatus to the cell surface, was found to be altered either through posttranscriptional regulation (51), or through loss-of-function mutations (44). In both cases, the lack of function of CD81 affected proper CD19 processing and surface presentation, leading to resistance to blinatumomab.

Lastly, loss of MHC I expression has been well documented as a key evasion mechanism to CD8+ cytotoxic T lymphocytes, and as a resistance mechanism to immunotherapy (33, 52). In a study of multiple myeloma patients treated with elranatamab, Friedrich et al. observed loss of MHC class I and BCMA surface expression on malignant plasma cells in some non-responder patients at relapse (53).

In the context of TCEs targeting intracellular antigens e.g. tebentafusp targeting a gp100 peptide presented on MHC I (18), the efficacy of TCEs is strictly dependent on the antigen presentation by MHC I molecules. Both soft (for example, epigenetic silencing and transcriptional downregulation), and hard (genetic mutations), loss of MHC I could lead to downregulation of surface antigen presentation (32), thus rendering the tumor cells resistant to TCEs treatment.

##### Alternative splicing

In B-ALL patients developing resistance to CD19 CART treatment, an alternatively spliced CD19 mRNA has been identified. Specifically, CD19 mRNAs skipping exon 2 led to N-terminally truncated CD19 variant lacking the binding epitope, and escaping killing by CD19 targeting CAR T cells (54). Interestingly, alternative splicing of CD19 with intraexonic splicing of exon 2 (termed CD19 ex2part) was also shown to lead to blinatumomab treatment failure at baseline or during treatment (44). Similarly, the alternative splicing of the 5’-UTR of CD20 mRNA led to a lower translational efficiency and enabled resistance to mosunetuzumab (55).

#### 2.1.3 Lineage switch

Lineage switch of cancer cells refers to the changes of their differentiation states to adopt characteristics of another lineage, evidenced by the expression of markers and functional properties typical of alternative lineage. B-ALL patients treated with CD19-directed CAR Ts displayed tumor cells switching from B cell lymphoid lineage to myeloid lineage (56–58), evidenced by the upregulation of myeloid marker CD33 and loss of B lymphoid lineage antigen CD19 (57). Similarly, B-ALL patients treated with blinatumomab also presented such a lineage switch (59). Lee et al. reported two patients who had experienced lineage switching after CD19-directed immunotherapies (case 1 by blinatumomab, and case 2 by CD19-targeting CAR-T cells), were salvaged with intensive myeloid-directed therapy, and experienced recurrent CD19+ B-ALL after clearance of the myeloid population (60). Remarkably, these two patients were then successfully rechallenged with blinatumomab. This study highlights the lineage plasticity and its implication on diligent therapy options (60). Mechanistically, Haddox et al. demonstrated that chromosomal aberrations led to rearrangement of KMT2A/AFF1 fusion protein, resulting in lineage switching from B-ALL to acute myeloid leukemia (AML), thus leading to resistance to blinatumomab treatment (59).

#### 2.1.4 Gene expression shift

A recent case report investigated the mechanism of acquired resistance to the DLL3 x CD3 TCE tarlatamab in a SCLC (Small Cell Lung Cancer) patient (61). The study found distinct transcriptional shifts between pre- and post-tarlatamab treatment tumors, with specific SCLC subtypes genes being upregulated or downregulated. This suggested a shift in gene expression profiles associated with resistance. Notably, NOTCH signaling pathway alterations were observed, with upregulation of NOTCH family genes and downregulation of DELTA-like family genes after treatment, supporting the hypothesis that changes in this pathway might contribute to resistance by affecting DLL3 expression.

#### 2.1.5 Post-translational modification

CD123, also known as interleukin-3 receptor alpha chain (IL3RA), is commonly expressed in certain hematologic malignancies including AML, making it a promising target for treating AML patients. Several CD123 x CD3 TCEs are being developed to direct T cells to CD123-expressing tumor cells (62). Through a CRISPR screen, deficiency in FUT8 (Fucosyltransferase 8) affecting core fucosylation of CD123, which is essential for the effective binding of CD123 x CD3 TCE was found to, lead to reduced binding affinity, impaired ability to engage T-cells and mediate tumor cell killing (63).

**Figure 4:**
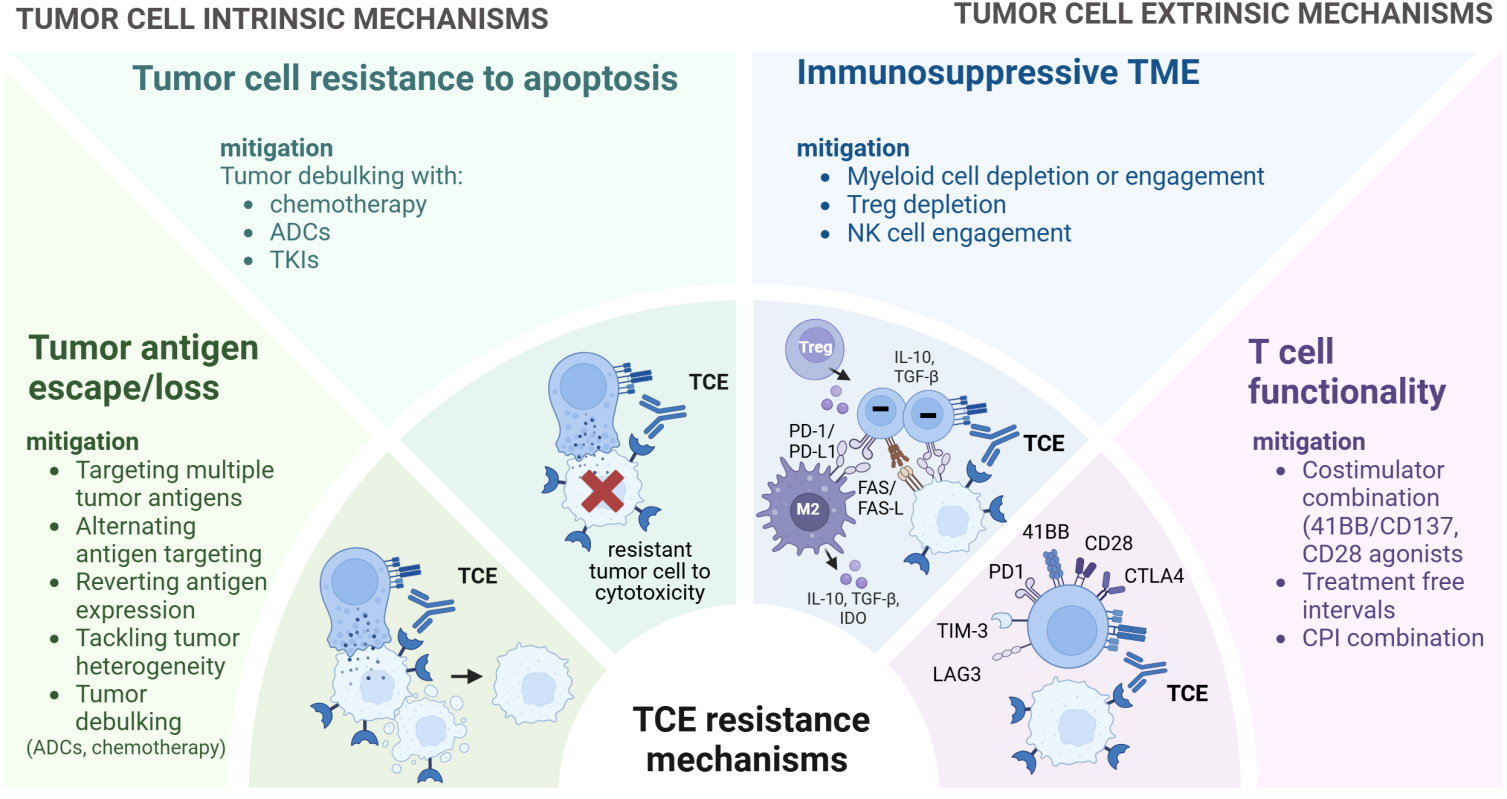
Strategies to overcome resistance to TCEs. Overview of strategies to overcome TCE resistance mechanisms. While certain strategies are currently under clinical investigation, others are preclinical and exploratory. These approaches encompass targeting multiple tumor antigens or alternating tumor antigen targeting, reverting antigen expression and preventing antigen loss. Integrating TCEs with other treatments such as chemotherapy, antibody-drug conjugates (ADCs) and Tyrosine Kinase inhibitors (TKIs) can facilitate tumor debulking and eliminate antigen-negative clones. Myeloid cell depletion or engagement, Treg depletion, NK cell engagement can help modulate the TME. Furthermore, to enhance T cell functionality, approaches such as costimulator combination, treatment free intervals, immune checkpoint inhibitors combination can be considered. TCE, T Cell Engager.

Together, multiple molecular mechanisms underlie tumor antigen downmodulation and escape/loss in both hematological malignancies and solid tumors, including both reversible (transcriptional or translational silencing, post-translational modifications), and irreversible (mutation-driven, genomic instability) mechanisms (**Figure 2**). The determinants guiding cancer cells to adopt one resistance mechanism over another remain largely unknown. However, emerging evidence suggests that the specific tumor antigen targeted by a TCE may critically influence the resistance pathways exploited by tumor cells. While antigens without a vital physiological role for tumor cells, such as CEA, can be lost, those essential for cancer cell survival, like HER2, must be maintained. Consequently, alternative resistance mechanisms evolve (50). To overcome these resistance mechanisms, it is key to develop strategies that can target multiple tumor antigens simultaneously to reduce the likelihood of single-antigen-driven tumor escape, or to restore target antigen expression/presentation where possible (**Figure 4**).

#### 2.1.6 Strategies to mitigate resistance mechanisms related to tumor antigens

##### Tumor Debulking to Tackle Tumor Heterogeneity

To overcome the challenge of tumor heterogeneity, potential strategies include the combination of TCEs with therapies or other modalities [e.g. chemotherapy or antibody-drug conjugates (ADCs)] that can reduce (debulk) tumor volume and aid in eliminating tumor antigen-negative clones. Furthermore, combining TCEs with tyrosine kinase inhibitors (TKIs) or radiotherapy could also be effective in addressing tumor heterogeneity and improving treatment outcomes.

##### Targeting of Multiple Tumor Antigens or Alternating Tumor Antigen Targeting

Dual- or multi-antigen targeting TCEs may help overcome the resistance mechanisms associated with tumor heterogeneity and treatment-induced antigen loss. A trispecific antibody targeting EGFR (epidermal growth factor receptor), EpCAM (epithelial cell adhesion molecule) and CD3 prevented colorectal tumor cell escape by tumor antigen loss, and importantly further delayed tumor growth and improved survival compared to the control bispecific antibody in preclinical models (64).

Beyond the design of multispecific TCEs that simultaneously target more than one tumor antigen on tumor cells, combining TCEs targeting different tumor antigens has proven to be a clinically relevant approach. The combination of BCMA- and GPRC5D-targeting TCEs, teclistamab and talquetamab, respectively, was recently shown to induce higher response rates and more durable responses than either therapy alone in a phase Ib-II study (65), substantiating the relevance of TCE-TCE combination approaches targeting different tumor antigens as a way to overcome resistance and improve TCE effectiveness (66–68). Of note, this approach may also come with challenges, including the limited space for targets whose expression is restricted to the tumor tissue, coming with a risk of on-target off-tumor toxicity.

##### Restoration of Antigen Expression or Prevention of Antigen Loss

Strategies to restore antigen expression include the use of small molecules and epigenetic modulation to upregulate tumor antigen expression on cancer cells. For example, DNA methyltransferase inhibitors have been shown to increase the expression of antigens in a study where a treatment-induced reduction of CEA levels (attributed to transcriptional silencing) was reversed by treatment with a DNA demethylating agent 5-Aza, which resensitized tumor cells to treatment with CEA-targeting TCE (50). Loss of CD20 upon treatment with the CD20 antibody rituximab was restored by 5-Aza (69–71), suggesting the potential of combining 5-Aza with CD20 targeting TCEs. Furthermore, Aurora Kinase inhibitor and Histone Deacetylases (HDACs) inhibitors have also been demonstrated to upregulate CD20 expression in preclinical models of lymphoma (72–74). Recently, combination of EZH2 inhibitor and HDAC inhibitor has been shown to upregulate CD20 expression in mosunetuzumab-treated preclinical models (75). Recently, the antibody drug conjugate polatuzumab vedotin was shown to result in upregulation of CD20 expression and synergize with mosunetuzumab in preclinical studies (76).

In instances where MHC I expression is downregulated through soft loss (for example, transcriptional downregulation, epigenetics silencing), the restoration of MHC I expression would be advantageous for the presentation of intracellular antigens (32). Treatment of HDAC inhibitors (77, 78), MEK (Mitogen-activated protein kinase kinase) inhibitor (79), and activation of double-stranded RNA sensor (80) have been shown to increase MHC I expression.

### 2.2 Tumor cell expression of immunomodulatory molecules

Cancer cell intrinsic resistance to TCE may additionally be mediated via immunomodulatory molecules, including the expression of checkpoint inhibitors, lack of expression of co-stimulatory molecules, and altered cancer-cell glycosylation (**Figure 2**).

The expression of checkpoint inhibitor PD-L1 on tumor cells impairs the activity of PD-1-expressing T cells. The role of PD-L1 in modulating T cell function upon TCE treatment first emerged as an immune escape resistance mechanism to blinatumomab (81, 82). This clinical observation (82) was consistent with *in vitro* investigation demonstrating that the expression of PD-L1 on ALL cells in the bone-marrow negatively impacted CD19 x CD3 TCE-mediated T cell cytotoxicity via the PD-1/PD-L1 axis. Alongside this, treatment with CD33 x CD3 TCE, FLT3 x CD3 TCE or BCMA x CD3 TCE induced PD-L1 expression on AML and multiple myeloma cells, respectively, which was found to negatively affect T cell cytotoxicity (83–85). This could be reverted by blocking the PD-1/PD-L1 interaction, which enhanced T cell cytotoxicity and tumor cell lysis (43, 86–90). Similarly, in Non-Hodgkin’s lymphoma, treatment with CD20 x CD3 TCEs induced PD-L1 expression on tumor cells, and the combination with PD-L1/PD-1 blocking antibodies, showed enhanced anti-tumor activity in preclinical models (91). The induction of PD-L1 expression on tumor cells by TCEs is inherently linked to their mechanism of action where T cell activation in the TME leads to IFN-γ release, a key regulator of PD-L1 expression (92, 93). Indeed, blocking IFN-γ signaling reduces TCE-induced PD-L1 upregulation (91).

For TCEs directed against solid tumors, PD-L1 expression on tumor cells was also found as a potential immune escape mechanism impairing T cell functions. In particular, Junttila et al. demonstrated that PD-L1 expression on target cells inhibited *in vitro* tumor killing by HER2 × CD3 TCE (94). They also showed that combining HER2 × CD3 TCE with a PD-L1 antibody enhanced T cell responses in CT26-HER2 tumors in huCD3 transgenic mice (94). It was also demonstrated that CEA x CD3 TCE treatment induces PD-1 and PD-L1 upregulation on T cells and of PD-L1 upregulation on tumor cells using *in vitro* and *in vivo* preclinical models (91, 95). Furthermore, the combination of CEA x CD3 TCE with a PD-L1 blocking antibody led to enhanced *in vivo* tumor growth inhibition (91). Additional preclinical studies further supported the use of PD-1/PD-L1 checkpoint inhibitors to enhance the activity of TCE directed against solid tumor antigens, including Trop-2 (96), CEACAM5 (91, 96), GUCY2C (97), as well as immTAC targeting NY-ESO-1 in NSCLC (98).

The strong rational and preclinical evidence of combining checkpoint inhibitors with TCE was subsequently evaluated clinically. Preliminary results from a phase 1 study showed that blinatumomab in combination with the PD-1 antibody nivolumab has a tolerable safety profile and achieved complete remission (CR) without minimal residual disease (MRD) in four of five patients with relapsed/refractory (r/r) B-ALL (NCT02879695) (99). Preliminary data from a phase I/II trial of blinatumomab with pembrolizumab also showed a tolerable safety profile, and achieved CR in two of four evaluable patients (NCT03160079) (100). In the solid tumor space, the combination of cibisatamab with the PD-L1 antibody atezolizumab was evaluated clinically. Although the efficacy readout was limited by adverse events as well as the formation of anti-drug antibodies against cibisatamab, clinical results suggested that atezolizumab improved overall rate survival over cibisatamab monotherapy (43, 101).

In addition to the expression of immune checkpoint molecules, cancer cell-intrinsic resistance to TCE therapy can also be linked by the lack of providing a co-stimulation signal. For instance, deletion or mutation of the co-stimulator CD58 has been observed in DLBCL (102), and relapsed Hodgkin lymphoma patients. Furthermore, genetic depletion of CD58 in tumor cells led to impaired TCE-mediated killing (103). Coherently, the same authors showed that concurrent abrogation of CD58 and CD80 co-stimulation led to decreased TCE potency (88). In other cases, co-stimulatory molecules are maintained or upregulated in cancer cells, and stimulation of this axis represents a successful strategy to boost TCE activity.

Altered cancer-related glycosylation recently emerged as a tumor escape mechanism. In the context of TCE-mediated resistance, the impairment of sialic acid on cancer cells increased the potency of several TCEs such as catumaxomab (EpCAM x CD3 TCE), and blinatumomab *in vitro* (2, 104).

Post-translational modifications such as glycosylation, phosphorylation, ubiquitination, acetylation, and palmitoylation impact PD-1/PD-L1 stability, localization, and their interactions with each other or with other proteins (105, 106). These modifications can influence immune checkpoint pathways and immune receptor signaling, ultimately affecting TCE resistance.

Lastly, tumors post tarlatamab treatment showed increased expression of T cell-related genes and immune markers, suggesting a shift towards an immunogenic tumor profile (61). Despite this, the efficacy of tarlatamab was reduced, indicating that resistance might be tackled by combining other immunotherapeutic strategies, such as targeting the SCLC-P (SCLC subtype signifying the transcription regulator POU2F3) subtype with PARP (poly(ADP-ribose) polymerase) inhibitors or IGF-1R, possibly alongside PD-1/PD-L1 inhibitors. The study also highlighted the need for clinically-feasible methods for SCLC subtyping to better understand and counteract resistance mechanisms to tarlatamab.

### 2.3 Tumor cell resistance to apoptosis induction

In some cases, tumor immune escape can arise from the insensitivity of tumor cells towards T cell-mediated cytotoxicity and subsequently apoptosis induction (**Figure 2**). Similarly to antigen-activated T cells, TCE-activated T cells release cytotoxic molecules, including perforin and granzymes, to induce killing of cancer cells (2, 107). Specifically, granzyme B enters the target cell through the pores created by perforin, initiates the apoptotic cascade by the cleavage of procaspase-3 to activate caspase-3 and cleavage of other substrates including BID (BH3 interacting-domain death agonist, a pro-apoptotic Bcl-2 family member), leading to mitochondrial outer membrane permeabilization and the release of cytochrome c, further propagating the apoptotic signal through the intrinsic pathway (108). Cancer cells can evade TCE-mediated killing through alterations of this cascade (McKenzie & Valitutti, 2023). For example, the serine protease inhibitor SERPINB9 has emerged as a critical granzyme B inhibitor. Knocking out SERPINB9 in cell lines increased their susceptibility to CD19-CAR and CD19 × CD3 TCE-induced cytotoxicity (109). In a separate study, Shen et al. demonstrated that knocking out BID (BH3 interacting-domain death agonist) in OVCAR8 cell lines impaired TCE-mediated apoptosis while the deletion of the two major anti-apoptotic genes CFLAR (CASP8 and FADD-like apoptosis regulator) and BCL2L1 (Bcl-2-like protein 1) enhanced TCE activity (103).

Although interference with perforin/granzyme-mediated apoptosis is widely recognized as a major tumor-intrinsic resistance mechanism to T cell-mediated cytotoxicity (110), resistance of tumor cells to TCE-induced killing may also arise from an alteration of tumor cell response to pro-inflammatory cytokines including TNF-α and IFN-γ, which play crucial roles in immune regulation, inflammation, and anti-tumor immunity. In an acquired resistance model to Her2 x CD3 TCE, tumor cells did not become resistant to T cell cytotoxicity via HER2 downregulation (as HER2 is required for cancer cell survival/proliferation), but rather via the downregulation of JAK2, which impaired tumor-intrinsic IFN-γ signaling (111). This is different from the previously-reported loss of CEACAM5 expression upon CEA x CD3 TCE treatment given that CEACAM5 is not required for survival/proliferation (50). Mutations affecting JAK1/2 have previously been described in the context of resistance to checkpoint inhibitor therapy (112, 113). Along this line, a CRISPR/Cas9 screening in AML identified JAK1 and PTPN2 as regulators of cancer cell resistance/sensitivity to CD123 x CD3 TCE (63). In addition, altered response to TNF-α was reported as a tumor immune evasion mechanism (114). Reverting cancer-intrinsic TNF-α signaling via SIK3 downregulation enhanced EpCAM x CD3 TCE-induced T cell killing (115).

In conclusion, tumor antigen dependent mechanisms such as antigen heterogeneity, antigen loss, lineage switch, post-translational modification, as well as expression of immune modulators and resistance to apoptosis can act in concert to limit the effectiveness of TCEs.

## 3 Tumor-extrinsic resistance mechanisms

### 3.1 Introduction to tumor microenvironment

The tumor microenvironment (TME) is a highly heterogeneous and dynamic system composed of diverse cellular, extracellular, and soluble components that collectively influence tumor progression and immune modulation (116, 117). The cellular constituents include malignant cells, cancer-associated fibroblasts (CAFs), and various immune cells, such as tumor-associated macrophages (TAMs), natural killer (NK) cells, myeloid progenitor cells, myeloid-derived suppressor cells (MDSCs), effector and regulatory T cells (Treg), dendritic cells (DCs), and neutrophils (**Figure 1**). The specific composition of these immune and stromal cells varies depending on tumor type, stage, and anatomical location (116). The extracellular matrix (ECM), composed of stromal cells, fibrous proteins, glycoproteins, proteoglycans, and polysaccharides, provides structural support and compartmentalization within the tumor, creating a physical and biochemical framework that regulates cell behavior and intercellular communication (118). Beyond its architectural role, the ECM actively modulates key processes such as cell differentiation, proliferation, and metastatic dissemination (119, 120). Additionally, secreted factors, including cytokines, chemokines, and various signaling molecules, are produced by both cancer and immune cells within the TME (121). These factors orchestrate immune cell trafficking, polarization, and functional modulation, thereby shaping the inflammatory status of the TME and influencing tumor immune evasion, progression, and response to therapy (122). The immunological profile and characteristics of the TME vary across different organs, necessitating consideration of organ-specific factors. For instance, the liver, which is intrinsically immunosuppressive, has been shown to induce a distinct suppressive program compared to the lung in metastatic disease models (123). These findings suggest that organ-specific immunosuppressive programming may have important clinical implications also for the activity of TCEs, as different disease sites within the same patient might require customized therapeutic strategies tailored to their unique microenvironment.

### 3.2 Immunosuppressive programming within TME

Immunosuppressive programming within the TME represents a broad network of cells and soluble mediators with key contributing factors consisting of a dense ECM, the presence of immunosuppressive immune cells (124, 125), the highly abnormal tumor vasculature (125), the expression of immunosuppressive molecules (96), and metabolic features including nutrient starvation, hypoxia, and acidity (126–129). Together, these elements hinder T-cell infiltration into tumors and compromise their functionality, thereby affecting their ability to sustain effective anti-tumor responses (130). As each of these components plays a critical role in shaping the immunosuppressive landscape of the TME, their distinct contributions to tumor progression, immune evasion, and therapeutic resistance to TCEs are elaborated in more detail in the following sections.

### 3.3 Physical barriers within TME

At a high level, the TME poses two major challenges to the efficacy of immunotherapies, including TCEs: it limits accessibility and it exerts immune suppression (131). The dense ECM within tumor stroma constitutes a physical barrier that restricts immune cell infiltration, leading to a scarcity of T cells available for TCE activation (131). Importantly, TME in solid tumors differs markedly from that in hematologic malignancies, with one of the primary distinctions being a dense, highly suppressive stroma, which poses challenges for T cell and immunotherapy access and activity. In contrast, hematologic malignancies such as leukemia and lymphoma, reside in the bloodstream and lymphoid tissues, which might enable easier access for T cells and TCEs. In solid tumors, TCEs must overcome physical barriers, including extravasation through the vasculature and penetration of the dense stromal matrix, before reaching the tumor cells (118). Consequently, targeting and modifying the stromal environment in solid tumors has shown potential in enhancing T cell-engaging therapies with studies demonstrating that an oncolytic adenovirus expressing an FAP-α x CD3 bispecific antibody effectively redirected T cell activity toward both cancer cells and fibroblast activation protein (FAP)-positive CAFs, resulting in improved anti-tumor responses (22, 132–134).

Furthermore, the tumor vasculature is often heterogeneous, leading to additional physical barriers to drug extravasation and sufficient T-cell infiltration (135, 136). The tumor vasculature is profoundly abnormal and characterized by structurally defective and leaky capillaries with impaired perfusion (137–139). The disorganized vasculature, together with the dense stroma, contribute to the development of hypoxic and acidic conditions within the TME, posing significant obstacles to the efficacy of cell- and immune-based therapies (140). The elevated interstitial pressure within solid tumors creates a significant physical barrier that hinders the penetration and distribution of anti-cancer therapies. Consequently, portions of the tumor mass may remain inaccessible, resulting in suboptimal treatment coverage and residual disease (141, 142). The combination of vascular abnormalities, high intra-tumoral pressure, and adverse metabolic conditions contributes to the complexity of delivering and sustaining effective immunotherapies in solid tumors.

### 3.4 Immunosuppressive cell populations within TME

Even when T cells successfully infiltrate the tumor, they encounter a hostile TME composed of immunosuppressive cell populations that include CAFs, Tregs, TAMs, MDSCs, tumor associated neutrophils (TANs), along with the inhibitory molecules they express (e.g. PD-L1), and the immunosuppressive factors they secrete (e.g., TGF-β, IL-10, indoleamine 2,3-dioxygenase (IDO), adenosine). Collectively, these stromal and immune cell populations, along with their secreted factors, establish a hostile and immunosuppressive milieu that facilitates tumor survival and metastasis, hindering effective anti-tumor immune responses, and fostering immunotherapy resistance (143–147).

CAFs play a key role in ECM remodeling, influencing tumor growth, immune exclusion, and metastasis (118, 148, 149). In addition, CAFs form a physical barrier that limits immune cell infiltration, particularly of cytotoxic CD8+ T cells, and secrete factors that promote angiogenesis, metabolic reprogramming, and immunosuppression (150–152). Subsequently, targeting CAFs has proven to be a valuable approach to enhance the potential of T cell-engaging therapies in solid tumors, as mentioned above (22, 132–134).

MDSCs and Tregs are major components that contribute to the immunosuppressive TME in solid tumors, reported to expand in several murine tumor models and to promote T cell dysfunction (153). Both mononuclear (M-MDSCs) and polymorphonuclear (PMN-MDSCs) subsets suppress anti-tumor immunity by inhibiting T-cell activation through PD-L1 expression, metabolic depletion of key nutrients such as cysteine, and secretion of immunosuppressive cytokines (154–156). Elevated MDSC levels are associated with poor responses to CAR T-cell therapy and tumor immune escape (157). Tregs, marked by CD4, CD25, and FoxP3 expression, suppress cytotoxic T-cell responses and contribute to immune tolerance within tumors (158, 159). Their accumulation in solid tumors correlates with poor prognosis, as they release cytokines such as IL-10 and TGF-β, further reinforcing the immunosuppressive nature of the TME (160–163).

In the context of TCE activity, the role of Tregs remains complex and controversial. Several studies reported that higher baseline levels of Tregs conferred primary resistance to TCEs and negatively correlated with clinical efficacy, leading to inferior clinical response and progression-free survival (PFS) (164–166). Other clinical studies reported a reduction (Foà et al., 2020) or a redistribution (167) of Treg cells upon blinatumomab treatment. Recent preclinical evidence found that BiTE treatment converted Treg function from immunosuppressive to immune-enhancing, contributing to antitumor activity in immunologically “cold” tumors (168). Since bispecific TCEs engage various T cell subsets, including Tregs, and direct their activity toward cancer cells, it is reasonable to hypothesize that Tregs do play a role in modulating TCE-mediated anti-tumor responses. However, the net effect of Tregs on TCE efficacy appears to be highly context-dependent, influenced by several factors such as the cellular composition of the TME, the abundance of cytotoxic T lymphocytes (CTLs) and conventional CD4+ T cells, as well as the effector-to-Treg ratio. Due to conflicting reports in the literature, further investigation is needed to clarify the specific conditions under which Tregs either support or impair the therapeutic potential of TCEs.

TAMs are actively engaged in promoting tumorigenesis and inhibiting antitumor responses through immunosuppression in the TME. TAM-mediated immunosuppression occurs through multiple mechanisms, including the release of anti-inflammatory cytokines (IL-10, IL-6, TGF-β, and prostaglandin E2 (PGE2), that restrict cytotoxic function of T lymphocytes and promote tumor growth), metabolic reprogramming (which promotes *Vegf* and *Arg1* expression by TAMs), expression of immune checkpoints (PD-L1, B7-H4, VISTA, which restrain the activity of tumor-specific effector T-cells) (169). Several strategies targeting pro-tumorigenic functions of macrophages have shown promising results in preclinical studies, and a few of them have also advanced to clinical trials (170). Approaches that deplete M2-macrophages or MDSCs have been shown to reverse the immunosuppressive TME of solid tumours and enhance anti-tumor efficacy of TCEs (171), underscoring the relevance of interfering with myeloid cells as an important approach to releasing TME immunosuppression (172, 173). Recently, M2-macrophages have been reported to inhibit tebentafusp -mediated tumor-killing, and in combination with IL-2, tebentafusp was reported to promote macrophage reprogramming to overcome their immunosuppression (174).

Beyond the extensively-reported immunosuppressive and pro-tumorigenic effects of macrophages, these cells can also play a crucial role in orchestrating anti-tumor immunity through their ability to secrete pro-inflammatory cytokines, phagocytose cancer cells and present tumor antigens to the cells of adaptive immunity (169). Recent studies reported the pro-inflammatory and anti-tumor contributions of macrophages in the context of TCE activity. Glofitamab-activated T cells, by virtue of secreting cytokines (TNFa, IFNg, IL-2, IL-8, and MIP-1b), rapidly activated neighboring monocytes, neutrophils, DCs, which amplified the inflammatory cascade initiated by T cells (175, 176). Combination of glofitamab with obinutuzumab (a CD20 type II glycoengineered IgG1 antibody) translated into rapid and more profound antitumor efficacy in preclinical studies (35). Mosunetuzumab therapy enhanced the capability of macrophages to perform antibody-dependent cellular phagocytosis (ADCP) and of NK cells to perform antibody-dependent cellular cytotoxicity (ADCC) (177), underscoring the potential of combining TCE-mediated T cell cytotoxicity with innate immune cell phagocytic and cytotoxic activity. Several macrophage-targeting strategies are currently in the preclinical stages or are being tested in clinical trials as a way to overcome resistance induced by TAM-mediated immunosuppression, with focus on depleting TAMs, preventing monocyte recruitment to the tumor, inhibiting macrophage polarization towards the M2 phenotype, re-educating polarized macrophage so that they can perform anti-tumor functions, or blocking inhibitory immune checkpoints (169). Due to the dynamic interplay between TCE-activated T cells and neighboring innate immune cells within the TME, resulting in the activation of the latter, combining TCE therapy with strategies targeting myeloid cells may enhance anti-tumor efficacy. Such approaches could involve engaging myeloid cells to harness their antibody-dependent cellular phagocytosis (ADCP) activity or depleting immunosuppressive myeloid populations to alleviate TME-induced immunosuppression. These strategies might be particularly relevant in solid tumors, where macrophages often represent the predominant and most ubiquitous cell type within the TME (91, 169, 178, 179).

The role of neutrophils in tumor immunology is complex, acting as a double-edged sword by contributing to both antitumor immunity and tumor-promoting activities (180). Homeostatic neutrophils are primarily involved in tumor elimination through mechanisms akin to their established anti-pathogen defense functions, supporting their role in immune-mediated tumor control. In contrast, protumorigenic activities are attributed to neutrophils that undergo transdifferentiation into immunosuppressive phenotypes in response to signals from the tumor microenvironment. Furthermore, neutrophils exert direct influences on multiple phases of tumor progression, contributing to both primary tumor growth and metastatic spread. Although experimental models in mice have consistently demonstrated that neutrophil depletion can significantly reduce metastasis formation (181–183), translating this approach into human therapy remains highly challenging due to the infection risk associated with neutropenia. Moreover, neutrophils are highly resilient cells (184), posing significant barriers to direct therapeutic targeting, with no effective anti-neutrophil agents currently available. Their robustness, coupled with their high plasticity in response to local tissue signals, complicates efforts to precisely modulate their activity in the tumor microenvironment. Consequently, the dual nature of neutrophils in cancer has generated an ongoing debate regarding their exact role and a ‘confusion’ on how they can be exploited therapeutically to enhance antitumor efficacy (180). To date, we have not identified any published studies specifically addressing the role of neutrophils in modulating the activity of TCEs. However, considering the abundance of neutrophils and their pivotal role in host defense and anti-tumor immunity, it is highly likely that these cells significantly influence TCE activity and potentially contribute to resistance mechanisms. Future investigations aimed at elucidating the role of neutrophils in the context of TCE therapy are highly anticipated and hold the potential to provide valuable insights into optimizing TCE efficacy.

### 3.5 Secreted factors and metabolic influence on the immunosuppressive TME

The TME is shaped by complex interactions between immune-modulating secreted factors and metabolic adaptations that drive tumor progression and immune evasion (126–129) (185) (126–129). Key transcription factors, NF-κB and STAT3, regulate the production of inflammatory cytokines such as TNF-α, IL-1, and IL-6, which recruit immunosuppressive cells, including TAMs and MDSCs (186–189). These cells, along with Tregs, secrete IL-10 and TGF-β, which suppress effector T cell responses and antigen presentation, further reinforcing immune escape (163). IL-10 impairs helper T cell activity, while TGF-β enhances tumor invasiveness and metastasis (190, 191).

Concurrently, metabolic competition in the TME deprives immune cells of glucose and glutamine, weakening their activation and cytotoxicity (192, 193). Tumor cells preferentially engage in aerobic glycolysis (Warburg effect), exacerbating CD8+ T cell dysfunction and promoting Treg expansion (194–197). Extracellular acidification, a hallmark of cancer, is primarily driven by the reliance of tumor cells on aerobic glycolysis to meet their metabolic demands, resulting in the coupled efflux of lactate and protons (194). This process is further amplified by the overexpression of enzymes such as carbonic anhydrase, which contributes to pH dysregulation in the tumor microenvironment (198). The resulting low pH profoundly impairs the function of multiple immune cell types, particularly effector CD8+ T cells, thereby weakening anti-tumor immunity and promoting immune evasion (127, 199–202). Excess lactic acid lowers TME pH, enhancing M2 polarization, suppressing NFAT-dependent T cell and NK-cell activation, and driving apoptosis of cytotoxic immune cells (196, 203–205). Additionally, lactate stabilizes FoxP3 via lactate dehydrogenase, reinforcing Treg-mediated immunosuppression (197). Hypoxia, an additional hallmark of tumors, further inhibits effector immune cells while promoting the accumulation of Tregs and M2-like macrophages (206–209).

Together, these cytokine-driven and metabolic mechanisms establish an immunosuppressive TME that facilitates tumor progression and resistance to immunotherapy.

### 3.6 T Cell functionality and exhaustion

As T cells are pivotal components in shaping the response to TCE, we report the phenotypical and functional analysis of T cells derived from patient material in hematological malignancies, including Multiple Myeloma and Non-Hodgkin’s Lymphoma, and elaborate on findings describing the role of T cell fitness in influencing the clinical response (**Figure 3**). Using an *ex vivo* restimulation assay with bone marrow patient samples, Verkleij et al. show that the baseline expression of PD-1 and HLA-DR on T cells as well as the presence of Tregs and bone marrow stromal cells correlated with reduced *in vitro* killing of multiple myeloma tumor cells by talquetamab (41). Along with this, findings from the MajesTEC-1 clinical study outline that responses to teclistamab are significantly influenced by the baseline characteristics of CD8+ T cells (67, 210–212). A higher baseline count of naive and memory CD8+ T cells was associated with improved responses, as these cells contribute to an active and less exhausted on-treatment immune profile. On the contrary, the presence of immunosuppressive Tregs correlated with poorer response. In addition, Van de Donk et al. reported higher Treg counts and lower CD8+ T cell counts as well as higher expression of PD-1, TIM-3, and CD38 on T cells from the peripheral blood and bone marrow from non-responder patients to teclistamab (213). Consistently, Friedrich et al. showed that the presence of specific T cell subsets, including CD8+ effector T cells in BMMC and peripheral blood of multiple myeloma patients is associated with better therapeutic responses to elranatamab (53). In contrast, a higher proportion of exhausted T cells correlated with poorer treatment response. In Non-Hodgkin’s lymphoma, for several CD20 x CD3 T cell engagers trends between clinical response and higher CD8+T cell infiltrates and lower Tregs count in biopsies collected prior treatment with the odronextamab, epcoritamab and glofitamab were reported (40, 214). Similarly, higher CD8+ T-cell levels were found in the peripheral blood from responders while higher prevalence of exhaustion markers including PD-1+, TIM-3+ and TIM-3+TIGIT+ were observed on CD8+ T cells from non-responders patients to epcoritamab (40, 214). Bröske et al. also reported that bulk RNA seq analysis of complete responder patient biopsies collected prior treatment with glofitamab exhibited an enrichment for gene transcripts associated with CD8+ T-effector phenotype while those of progressing disease patients exhibited a high PD-1 signature (38). Beyond the role of baseline T cell states in determining TCE treatment outcomes, several studies have also evaluated how repeated TCE treatment cycles may influence T cell activity, examining effects on T cell phenotype and functionality in peripheral blood collected longitudinally during treatment. Philipp et al. investigated the effects of blinatumomab treatment on T cell function and exhaustion. Using both *in vitro* preclinical models and *ex vivo* patient samples, they showed that blinatumomab led to progressive T cell exhaustion, as marked by reduced T cell cytotoxicity and upregulation of PD-1 and TIM-3 exhaustion markers over time (215). The introduction of treatment-free intervals could counteract exhaustion induced by chronic exposure to blinatumomab (215). In line with these findings, Verkleij et al. reported progressively-diminished T cell functions, including proliferation and cytotoxicity when restimulating T cells in multiple myeloma patients treated with talquetamab or teclistamab (53, 216). Of note, T cell cytotoxicity was mostly impacted at the time of progressing disease and remained only partially reduced after 14 cycles of treatment. These studies suggest that T cell functions may be partially impaired at the later cycles of treatment, suggestive of an adaptive immune resistance. Altogether the phenotypical and functional analysis of patient material suggest that the presence of exhausted T cells and immunosuppressive T regs in the tumor microenvironment and peripheral blood of lymphoma and multiple myeloma patients negatively affects TCE outcome, whereas the presence of naive/effector T cells correlates with better response to TCE. Combination with co-stimulatory molecules represents a promising approach to restore T cell function and overcome T cell exhaustion which may be either present before TCE treatment or induced by prolonged exposure to TCE (217–219).

In preclinical models, the combination of TCEs targeting solid tumor and hematological surface antigens showed synergistic activity when combined with complementary tumor-targeted CD28 bispecific antibodies. For example, the CD19-targeted CD28 costimulator enhanced T cell function and *in vivo* activity in DLBCL models when combined with glofitamab (220). Additionally, 4-1BB agonists enhance T cell expansion and persistence by potentiating CD8+ T cell cytotoxic activity and supporting memory T cell formation. A notable advancement includes FAP-4-1BBLas well as CD19-4-1BBL (englumafusp alfa) constructs, which selectively deliver co-stimulation to the tumor microenvironment, minimizing off-target toxicities while boosting local T cell responses when combined with cibisatamab and glofitamab, respectively (91, 219). Based on this, the combination of glofitamab and CD19-CD28 was evaluated in a phase 1 dose escalation study (NCT05219513) and the combination of glofitamab with englumafusp alfa is currently evaluated in a randomized clinical phase 2 trial (NCT04077723). Similarly, REGN5837, a bispecific antibody which cross-links CD22-expressing tumor cells with CD28-expressing T cells, enhanced the activity of the odronextamab in DLBCL humanized *in vivo* models by potentiating T cell activation and cytolytic function (221). These studies led to the current clinical evaluation of the combination of odronextamab and REGN5837 (NCT05685173). Furthermore, recent preclinical experiments provide rationale for the triple combination with CD19-CD28 and CD19-41BB ligand to further prolong the response to glofitamab in the aggressive DLBCL OCI-Ly18 model in humanized NSG mice (220).

### 3.7 Challenges of TCE development in solid tumors

Despite the remarkable success of T cell engagers in hematologic malignancies, and extensive research aimed at extending their efficacy to solid tumors, their clinical impact in solid cancers remains limited. The unique challenges of TCE therapy in solid tumors stem from physical and immunological barriers within the TME, which limit tumor accessibility and T cell Infiltration, tumor antigen heterogeneity, and T cell exhaustion, all of which limit efficacy compared to hematologic malignancies.

#### 3.7.1 Tumor Accessibility and T-Cell Infiltration:

In hematologic malignancies, TCEs have direct access to circulating tumor cells in the bloodstream, or an easier access to the same in lymphoid tissues, which facilitates tumor killing. In solid tumors, physical barriers such as dense ECM, stromal fibrosis, abundant desmoplastic reaction, and abnormal vasculature prevent efficient T cell trafficking and penetration into the tumor core, limiting therapeutic efficacy as outlined above (94, 117, 120, 121, 222–226).

#### 3.7.2 Immunosuppressive TME

Even when T cells make it to the tumor, they rapidly encounter a hostile TME, particularly in solid tumors. The accumulation of Tregs, MDSCs, TAMs, TANs and CAFs create an immunosuppressive TME, which rapidly inhibits T cell activation and cytotoxic activity through cytokine secretion, metabolic competition (227, 228), physical barriers and direct cellular contacts. In contrast, hematologic malignancies, while also exhibiting immune evasion mechanisms, lack a physical TME barrier, allowing T-cell engagers to engage target cells more effectively (229).

#### 3.7.3 Antigen Expression and Heterogeneity

Hematologic malignancies often express well-defined, lineage-specific antigens (e.g., CD19 in B-cell malignancies), which are largely restricted to tumor cells, minimizing off-target toxicity (230). Solid tumors, however, exhibit greater antigen heterogeneity and often express target antigens on both malignant and normal tissues, increasing the risk of on-target, off-tumor toxicity (24, 28–30, 231).

#### 3.7.4 Persistence and T Cell Exhaustion:

In solid tumors, chronic antigen exposure, hypoxia, and metabolic stress contribute to rapid T cell exhaustion, reducing cytotoxic efficacy (232–234).

#### 3.7.5 Cytokine Release and Toxicity:

Cytokine release syndrome (CRS) is a major concern in both settings, but it is more manageable in hematologic malignancies due to the more restricted tumor antigen expression, and ability to control T cell activation via optimized dosing strategies, steroid treatment or IL-6 blockade (Tocilizumab) (235). In solid tumors, the expression of targeting antigen in normal tissues leads to on-target-off-tumor toxicity, increasing cytokine release and causing normal tissue damage, which limits therapeutic window and restricts clinical benefit. Taken together, both solid tumors and hematological malignancies present a unique set of challenges, which are, however, more pronounced in solid tumors. Overcoming these obstacles requires strategies such as enhancing T cell infiltration, reducing immunosuppression, and optimizing tumor antigen targeting to improve therapeutic outcomes, as outlined in the future perspectives section.

### 3.8 Knowledge gaps and limitations of preclinical models

One of the key limitations in current research - and a hurdle to improving the clinical success rates of TCEs - is the limited predictive value of preclinical models. While *in vivo* murine models, *ex vivo* patient tumor explants, and *in vitro* human tumor organoids provide valuable mechanistic insights, they fail to fully recapitulate the complexity of the human TME, including long-term tumor-stroma interactions, immune evasion mechanisms that evolve over years or decades, immune cell dynamics, and interpatient heterogeneity (236–245). As such, many TCEs demonstrating promising preclinical efficacy have failed in clinical trials due to unanticipated toxicities, suboptimal efficacy, or an inability to overcome TME-imposed immunosuppression. Murine models, despite their utility in evaluating TCE pharmacokinetics, biodistribution, and tumor targeting, exhibit species-specific differences in antigen expression, which limit their ability to predict on-target, off-tumor toxicity. For instance, while human EGFR is broadly expressed across epithelial tissues, murine EGFR expression varies significantly, leading to an underestimation of toxicity risks in preclinical studies (246). Similar discrepancy has led to adverse events in clinical trials with HER2- and CEA-targeted therapies (43, 247, 248). The development of human tumor antigen transgenic (hTA Tg) mice, which accurately mimic human tumor antigen (TA) expression, could represent a significant advancement in improving the translational relevance of preclinical models.

Beyond limitations in TA expression, murine models have fundamental species differences in immune cell composition, cytokine networks, T cell activation and exhaustion pathways, which do not accurately reflect human immune responses (236, 249–251). Even advanced humanized mouse models, such as stem cell-humanized mice, which aim to recreate human immune responses, fail to fully replicate a functional human immune system (237, 252). While these models support human T and B cell activity, they often lack - or have suboptimal numbers or function of human myeloid cells (which are important contributors to TCE resistance in patients), and of NK cells, and retain murine stromal and vascular components. Continued investment in the development of next-generation humanized mouse models, that fully recapitulate the phenotype, number, and functionality of human immune cells (253–256), combined with crossing them with hTA Tg mice (to mimic human tumor antigen expression, as above), or with hFcRn mice (to better reflect human antibody pharmacokinetic properties), can enhance the predictive validity of preclinical models. Additionally, integrating patient-derived xenografts (PDXs), which better reflect tumor and antigen heterogeneity in efficacy studies, can improve the reliability of preclinical assessments in predicting the clinical efficacy of TCEs.

Further to efficacy and safety aspects, several TCEs are engineered using non-human sequences, making them susceptible (despite humanisation efforts) to anti-drug antibody (ADA) formation in patients (257–259). In clinical settings, ADA formation can neutralize TCE activity, alter pharmacokinetics, and increase the risk of hypersensitivity reactions, posing a significant barrier to long-term therapy success. Due to the different mechanisms underlying antibody response, conventional animal models are less likely to predict clinical incidence of immunogenicity (260, 261). Emerging artificial lymphoid organoids, and additional screening tools, offer the potential to model B- and T-cell interactions *in vitro*, providing a more accurate prediction of ADA formation and chronic immune responses (262, 263). Finally, integrating multi-omic screening for HLA-binding epitopes and immune receptor profiling may enable the early identification of immunogenic TCE regions before clinical trials (264–266).

More advanced *in vitro* and *ex vivo* models used for preclinical evaluation of TCEs, including patient-derived organoids (PDOs) or primary tumor explants (*ex vivo* cultured tumor slices or fragments), preserve tumor heterogeneity and allow co-culturing with patient-derived immune cells, enabling TCE selectivity and immune activation assessment in a human-relevant system. However, they remain limited in modeling immune infiltration, vascularization, and systemic factors (243, 267), and suffer from poor *ex vivo* viability of primary tumors and are thus limited to short-term experiments. The advancement of these models, to integrate key features such as vascularization, immune cell recruitment and infiltration, along with the optimization of *ex vivo* culturing conditions, and the development of platforms that support long-term *ex vivo* experiments, will significantly enhance their translational relevance and use. These improved models will better mimic the human TME and provide a complementary approach to existing *in vivo* systems, ultimately refining the predictive value of preclinical testing for TCE efficacy, resistance mechanisms, and safety assessments (243, 267, 268).

Lastly, a critical limitation in the field is the lack of robust biomarkers for predicting TCE resistance and patient response. While factors such as antigen density, T cell state and exhaustion markers, along with cytokine profiles, have been explored, no clinically validated biomarker reliably stratifies patients who will benefit from TCE therapy versus those at risk of resistance, hindering precision medicine approaches (269). Further investment in reverse translation - integrating insights from clinical samples and iteratively applying key findings into preclinical development - is essential for continued optimization of TCE formats, designs, and combination strategies. This approach is particularly important in the current landscape, following the recent approvals of several TCEs in hematologic malignancies and the early success of a few in solid tumors. Continuous refinement of preclinical models and therapeutic strategies based on clinical data will be pivotal in enhancing efficacy, mitigating safety profiles, and overcoming resistance, ultimately driving more effective TCE translation into clinical practice.

In summary, to overcome current translational challenges and improve the clinical success rates of TCEs, particularly in solid tumors, next-generation preclinical models must better replicate human tumor-immune interactions to enhance predictive validity. By leveraging multi-platform strategies, including humanized mouse models, human tumor antigen transgenic mice, patient-derived xenografts, and advanced *in vitro/ex vivo* organoid/tumor explant systems, future preclinical models can better capture toxicity risks, immune responses, and resistance mechanisms, ultimately enhancing the safety, efficacy, and clinical applicability of TCE therapies.

## 4 Future perspectives

As TCEs are increasingly used in clinical settings to treat hematological malignancies and, to a growing extent, solid tumors, both primary resistance and treatment-induced resistance leading to relapse are becoming more common. The high potency of TCE-mediated tumor cell killing exerts significant evolutionary pressure on tumors to evade T cell recognition. Over the past years, preclinical studies and *ex vivo* analyses of patient samples have revealed a diverse range of resistance mechanisms, involving both tumor cell-intrinsic and tumor cell-extrinsic factors that influence the tumor microenvironment. Unlike resistance to targeted therapies such as kinase inhibitors directed at tumor drivers, resistance to TCEs is complex, multifactorial, and can arise at various stages of treatment. Tumors often exploit mechanisms originally designed to suppress immune responses and protect normal tissues from excessive damage—many of which have also been identified as resistance mechanisms to checkpoint inhibitors (113, 152).

Overcoming resistance to TCEs requires a multifaceted approach, necessitating the development and evaluation of novel strategies in clinical trials. Combination therapies are a key focus, including the use of TCEs alongside cytotoxic treatments such as chemotherapy and antibody-drug conjugates (ADCs) (270, 271) for tumor debulking, as well as dual-targeting TCEs designed to prevent the escape of tumor antigen-negative clones (64) (**Figure 4**). Avidity-driven dual targeting approach offers potential for enhanced tumor selectivity and diminished on-target-off-tumor toxicity, enabled by the concomitant co-expressed of antigens on tumor cells but not in healthy tissues.

Another promising strategy involves combining bispecific TCEs with approaches that modulate the TME to counteract stroma-induced immunosuppression and enhance TCE-driven antitumor activity. Integrating TCEs with complementary immunotherapies—such as immune checkpoint inhibitors (e.g., PD-1/PD-L1 antibodies), co-stimulatory receptor agonists (e.g., 4-1BB, CD28, CD2), or cytokine-based therapies (e.g., IL-2, IL-7, IL-18)—offers significant potential for improving treatment efficacy, particularly in solid tumors (**Figure 4**). As treatment with immune checkpoint inhibitors and TCE come with a risk of immune related adverse events, including colitis and CRS, combinatorial approaches to overcome resistance may require a thorough clinical development and safety monitoring.

With the remarkable clinical progress with recently-approved TCEs in hematological malignancies, and growing evidence supporting their efficacy in solid tumors, these therapies are set to become a cornerstone treatment for various tumor types. To fully realize their transformative potential, it is imperative to continue investing into unraveling mechanisms underlying TCE resistance, develop them using next-generation, translationally-relevant preclinical models, and select optimal combination partners to foster activity and durability of response. Addressing these challenges will not only enhance therapeutic efficacy but also accelerate the evolution of TCE-based therapies in redefining cancer treatment paradigms.
